# Chest Movement and Respiratory Volume both Contribute to Thoracic Bioimpedance during Loaded Breathing

**DOI:** 10.1038/s41598-019-56588-4

**Published:** 2019-12-27

**Authors:** Dolores Blanco-Almazán, Willemijn Groenendaal, Francky Catthoor, Raimon Jané

**Affiliations:** 1 Institute for Bioengineering of Catalonia (IBEC), The Barcelona Institute of Science and Technology, Baldiri Reixac 10-12, 08028 Barcelona, Spain; 2 Universitat Politècnica de Catalunya · BarcelonaTech (UPC), Barcelona, Spain; 3 Biomedical Research Networking Centre in Bioengineering, Biomaterials and Nanomedicine (CIBER-BBN), Barcelona, Spain; 4 imec the Netherlands/Holst Centre, High tech campus 31, 5656AE Eindhoven, The Netherlands; 5 imec, Heverlee, 3001 Belgium; 6 KU Leuven, Heverlee, 3001 Belgium

**Keywords:** Health care, Diagnosis

## Abstract

Bioimpedance has been widely studied as alternative to respiratory monitoring methods because of its linear relationship with respiratory volume during normal breathing. However, other body tissues and fluids contribute to the bioimpedance measurement. The objective of this study is to investigate the relevance of chest movement in thoracic bioimpedance contributions to evaluate the applicability of bioimpedance for respiratory monitoring. We measured airflow, bioimpedance at four electrode configurations and thoracic accelerometer data in 10 healthy subjects during inspiratory loading. This protocol permitted us to study the contributions during different levels of inspiratory muscle activity. We used chest movement and volume signals to characterize the bioimpedance signal using linear mixed-effect models and neural networks for each subject and level of muscle activity. The performance was evaluated using the Mean Average Percentage Errors for each respiratory cycle. The lowest errors corresponded to the combination of chest movement and volume for both linear models and neural networks. Particularly, neural networks presented lower errors (median below 4.29%). At high levels of muscle activity, the differences in model performance indicated an increased contribution of chest movement to the bioimpedance signal. Accordingly, chest movement contributed substantially to bioimpedance measurement and more notably at high muscle activity levels.

## Introduction

Respiratory diseases are diagnosed and monitored by measuring the patients’ pulmonary function. Spirometry is the main test for assessing many respiratory diseases such as asthma or chronic obstructive pulmonary disease^1^. Spirometry requires the use of facemasks or mouthpieces^2^ which usually are obtrusive and uncomfortable for the patients. In addition, this equipment could modify their breathing^3^. Lately less invasive methods are investigated as an alternative to classical methods to provide a continuous monitoring although more evidence for clinical application is needed^4^. One of these alternatives is thoracic bioimpedance which measures impedance changes over time. Thoracic bioimpedance has been widely studied as a non-invasive technique for measuring respiration, and several studies have shown a linear relationship with respiratory volume^5–11^. However, not only airflow contributes to the measured thoracic bioimpedance measurement, but it is a combination of the impedance of several body tissues, organs and fluids in this zone. Defining how all thoracic components contribute to the measurement is unclear. Modeling all these components as an electronic circuit can be difficult. Alternatively, previous studies presented computer simulations of these contributions in finite element human thorax models^12–14^. These simulations studied different electrode locations and showed that electrodes positioned around the middle of the thorax reflect changes in bioimpedance of the lungs^12^.

Early studies included animal testing to explain other changes in thoracic bioimpedance than the changes resulting from respiratory volume. These studies analyzed the relationship of bioimpedance relationship with respiratory volume and thoracic diameter^15,16^. Baker *et al*. showed that thoracic circumference or diaphragm displacement produced components of bioimpedance that combine linearly for normal volumes and probably nonlinearly for extreme conditions^15^. In addition, recent studies indicated that during abnormal breathing the relation between bioimpedance and volume appeared non-linear^8,10^. This apparent non-linearity could be caused by the contribution of other components to the bioimpedance measurement. Thus, thoracic bioimpedance changes seem to be a combination of volume and other thoracic changes but both the ratio and how these contributions change over different breathing types are unclear.

Inspiratory threshold loading enables the study of inspiratory muscle function and was used previously in respiratory studies^17–20^. Imposing inspiratory loads requires increased breathing pressure and are associated with breathing pattern changes and diaphragm fatigue^17,18^. In previous studies, we studied the relationship between bioimpedance and respiratory volume during inspiratory loaded breathing^11,21^. The analysis of the temporal relation between the signals revealed delays when loads were imposed^21^. These differences in time could be related to differences in thoracic breathing movement and displacement as a result of the increase of the breathing effort and changes in breathing pattern.

The aim of the current study is to investigate the relevance of respiratory volume and chest movement contributions, represented by a spirometer and an accelerometer respectively, to thoracic bioimpedance measurement. Therefore, the objective is to have more knowledge about thoracic bioimpedance changes and its relation with breathing movement and respiratory volume. The combination of accelerometer and bioimpedance measurements has been used before, however, these studies used the combination for motion aritifact removal^22,23^. These studies did not examine the relationship between these signals. We hypothesized that bioimpedance changes are a combination of breathing movement and airflow and that these contributions can change as a function of muscle force used for breathing. Consequently, we measured airflow, thoracic bioimpedance and accelerometer data to study the relation of thoracic bioimpedance with volume and chest motion. This relation was studied though linear mixed-effect models and neural networks by reconstructing bioimpedance signals for different levels of muscle activity as a result of an inspiratory threshold loading protocol. Therefore, the novelty of our study compared to both the literature and our own earlier work is the inclusion of chest movement in the analysis of bioimpedance changes during loaded breathing. The conclusive results of this study will contribute to better understand of thoracic bioimpedance signal and to reinforce its application for non-invasive respiratory monitoring.

## Methods

### Subjects

The study included ten healthy non-smoker subjects (4 females) of age 24–37 years (mean 30.5) and body mass index 19.5–26.8 kg m^−2^ (mean 23.1). None reported any respiratory disease.

### Ethical approval

The research was approved by the Institutional Review Board of the Institute of Bioengineering of Catalonia and followed the World Medical Association’s Declaration of Helsinki on Ethical Principles for Medical Research Involving Human Subjects. The subjects were informed about the measurements and protocol procedure and provided their informed consent before participation.

### Respiratory protocol

The study consisted of performing an incremental inspiratory threshold loading protocol during physiological signal measurements. During this kind of protocol, inspiratory loads are imposed to the subjects who need to increase the pressure to breath completely. The subjects wore a nose clip to prevent nasal breathing and were comfortably seated in upright position during the measurements.

**Figure 1:**
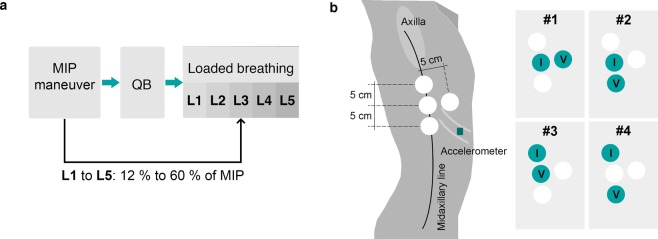
Sensors location and protocol description of the presented study. (**a**) The respiratory protocol includes three steps: MIP manuever^24^, recording of quiet breathing (QB) during 2 min and loaded breathing. The loaded breathing consisted of thirty breaths while an inspiratory threshold load was imposed (progressively selected from 12% to 60% of the subject’s MIP) (**b**) Representation of the four tetra-polar electrode configurations and accelerometer locations. The electrode configurations were symmetric from the midsternal line. #1, #2, #3 and #4 denote the electrode configurations where the used electrodes are highlighted, *I* refers to the injecting electrodes and *V* to the voltage measurement electrodes. The accelerometer was placed on the subjects’ skin approximately over the anterior axillary line and along the seventh or eighth intercostal space.

The inspiratory threshold loading protocol used in the presented study consisted of imposing five incremental inspiratory threshold loads to the subjects while breathing. The inspiratory threshold values were increasing percentage values from each subject’s maximal static inspiratory pressure (MIP) from functional residual capacity. The MIP was obtained from each subject by performing a maximal volitional manoeuvre^24^. After the maximal manoeuvre, the subjects’ quiet breathing (QB) was recorded for 2 minutes and after that, the subjects breathed while inspiratory loads were imposed. The five load thresholds were progressively selected from 12% to 60% of the subject’s MIP (L1, L2, L3, L4 and L5). Each load included 30 breaths and was followed by a resting period to return to baseline (Fig. 1a).

The MIP maneuver and inspiratory loads were imposed using a class 1 medical inspiratory muscle trainer (POWERbreathe KH2, POWERbreathe International Ltd, Southam, UK)^25^. The device controlled electronically the threshold resistances imposed to the subjects.

### Measurements

The physiological data were acquired by a wearable research prototype device (Stichting imec The Netherlands) and a standard wired acquisition system (MP150, Biopac Systems, Inc. Goleta, CA, USA).

Bioimpedance was measured at four tetrapolar electrode configurations simultaneously using the wearable device. The device measures isolated bioimpedance values from the four configurations by switching the current injection and the voltage measurement (MUSEIC v1 chip, Stichting imec The Netherlands)^26^. The four electrode configurations were previously presented^11^ and are represented in Fig. 1b. The electrodes configurations were symmetric from the midsternal. Configuration #1 was horizontal, the injecting current electrodes were placed at 7 cm from the axillas on the midaxillary lines and the voltage electrodes were at 5 cm away from the injecting ones closer to the midsternal line. Configurations #2, #3 and #4 were verticals and all the electrodes were placed on the midaxillary lines. The electrodes of configurations #2 and #3 were separated 5 cm but electrodes of configuration #3 were on a upper zone. Configuration #4 covered a broader zone because the electrodes were separated 10 cm. For all the vertical configurations, the voltage electrodes were the lower ones. We included four electrode configurations to evaluate if the differences in geometry and distances were relevant to our analysis. The amplitude of the injection current was 110 *μ*A at 80 kHz.

Respiratory airflow and accelerometer data were recorded using the Biopac wired acquisition system. Airflow was acquired with Biopac transducer (pneumotach transducer TSD107B, Biopac Systems, Inc.). The airflow transducer were connected to a differential amplifier which amplified 1000 times and low-pass filtered (*fc* = 300 Hz) the signal. Disposable mouth pieces with bacterial filters were attached to the pneumotach and the subjects breathed through them.

Accelerometer data was measured with a tri-axial accelerometer (TSD109C2, Biopac Systems, Inc.) connected to its associated interface (HLT100C, Biopac Systems, Inc.). The accelerometer was placed on the subjects’ skin with adhesive rings close to the lower bioimpedance electrodes (Fig. 1b), approximately over the anterior axillary line and along the seventh or eighth intercostal spaces^27^. The location was selected to measure the movement of approximately the same area covered by thoracic bioimpedance measurement.

Electrocardiogram (ECG) was recorded across the wearable device and the wired Biopac system (ECG100C, Biopac Systems, Inc.) and was used to synchronize the signals from both systems.

The signals acquired by Biopac amplifiers were A/D converted by Biopac MP150 system with a sampling rate of 10 kHz. The wearable device acquired the bioimpedance and ECG signals at sampling rates of 16 Hz and 512 Hz respectively.

Stress test Ag/AgCl electrodes (EL501, Biopac Systems, Inc.) were used in bioimpedance and ECG measurements.

### Signal processing

#### Signals synchronization

The signals from the systems were synchronized using the ECG signals. The delays between systems were computed as the lag that maximizes the cross-correlation of the ECG signals and subsequently they were corrected in the signals.

#### Signal filtering

The four bioimpedance channels were high-pass filtered to reduce the baseline oscillations (zero-phase 4^*th*^ order Butterworth, *fc* = 0.05 Hz). The sampling frequency was increased from 16 Hz to 200 Hz by cubic interpolation to improve the time resolution.

Accelerometer and airflow signals were low-pass filtered to avoid aliasing (8^*th*^ order Chebyshev Type I, *fc* = 80 Hz) and resampled from 10 kHz to 200 Hz. The accelerometer data, denoted as *acc*, was measured by a tri-axial accelerometer and consisted of three signals corresponding to the acceleration in the three spatial directions (*acc*_*X*_, *acc*_*Y*_ and *acc*_*Z*_) related to the sensor axes. The accelerometer orientation was the same for all the subjects, hence, the axes approximately represent the same spatial direction over subjects. Airflow signal was low-pass filtered (zero-phase 4^*th*^ order Butterworth, *fc* = 5 Hz) to remove the high frequency content not related to the breathing. The respiratory volume were computed by trapezoidal numerical integration of the low-pass filtered airflow signal.

Bioimpedance, *acc* and volume signals were low-pass filtered (zero-phase 4^*th*^ order Butterworth, *fc* = 1 Hz) and high-pass filtered (zero-phase 4^*th*^ order Butterworth, *fc* = 0.05 Hz) to get the respiratory information. The *acc* signals filtered in this low frequency range provide information of the surface chest motion, denoted as *acc*_*CM*_, and particularly for each accelerometer axis denoted as $ac{c}_{C{M}_{X}}$, $ac{c}_{C{M}_{Y}}$, and $ac{c}_{C{M}_{Z}}$. For simplicity we are going to use *acc*_*CM*_ notation for the three accelerometer signals related to chest movement. The signals were normalized in the range of [−1, 1] for each subject.

#### Muscle force estimation

Surface mechanomyography measured by accelerometers on the chest wall over the lower intercostal spaces (sMMG_*lic*_) has been suggested to be able to provide a noninvasive index of inspiratory muscle force. We computed this index from our accelerometer signals based on the study of Lozano-García *et al*.^20^. Consequently, the *acc* signals were low-pass filtered (zero-phase 4^*th*^ order Butterworth, *fc* = 35 Hz) and high-pass filtered (zero-phase 4^*th*^ order Butterworth, *fc* = 5 Hz) to get information of the muscle fibre vibration (sMMG_*lic*_). Note that we used the data from the same accelerometer but the bandwidth used in the muscle force estimation is different from the chest movement one. We got the total acceleration of the muscle fibre vibration (|sMMG_*lic*_|) by computing the Euclidan norm of the three signals. The envelope of the resulting signal was computed as the root mean squared (RMS) values in windows of 750 ms and 90% of overlap^20^. We computed the muscle force estimation as the mean value of the RMS |sMMG_*lic*_| cycle by cycle.

#### Respiratory cycles selection

All the signals were fragmented in respiratory cycles using a thresholding algorithm applied to the airflow signal^28^. The respiratory cycles that were affected by artefacts were rejected. We determined the rejected cycles as the cycles which the |sMMG_*lic*_| maximum value was higher or lower than three times the scaled median absolute deviation of the cycle maximum values of each subject and load. Only 98 respiratory cycles were rejected (5.35% of the total).

### Contribution analysis to thoracic bioimpedance

**Figure 2:**
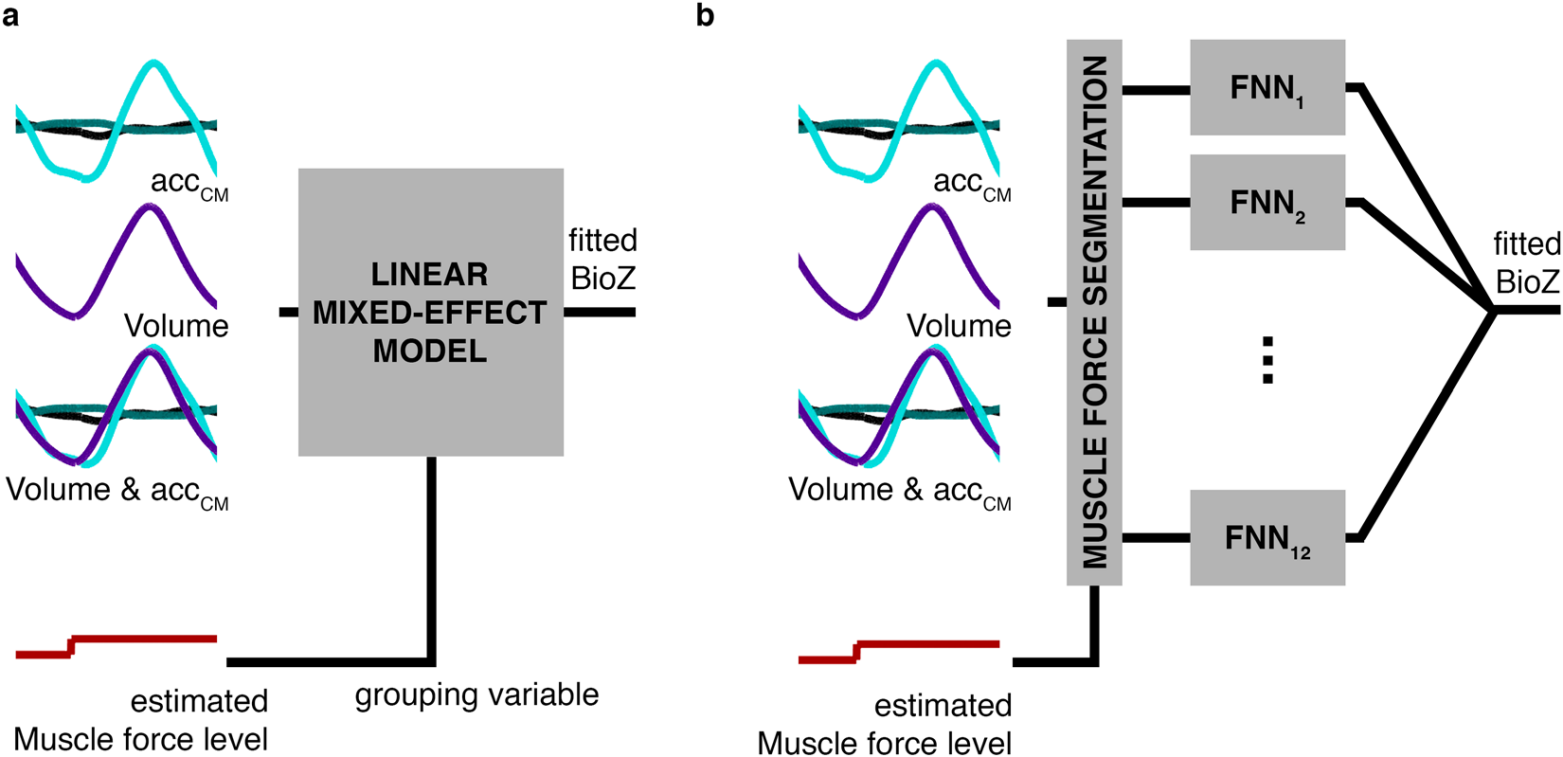
Contribution analysis for each subject and electrode configuration. (**a**) Linear mixed-effect models were used to evaluate linear characterization of bioimpedance. Three different models were computed by changing the predictor variables: *acc*_*CM*_, volume and both volume and *acc*_*CM*_. (**b**) Feedforward neural networks (FNN) were used to evaluate non-linear characterization of bioimpedance. Three combinations of inputs were used: *acc*_*CM*_, volume and both volume and *acc*_*CM*_. We computed one FNN for each level of muscle force estimation. In this figure, BioZ is used as contraction of bioimpedance term.

We studied the relevance of volume and chest movement contributions to the measured bioimpedance signal using linear mixed-effect models and neural networks (Fig. 2). The objective of our work was to understand the changes of the bioimpedance signal at different levels of muscle force. At this stage our aim was not to be able to predict the signal. Therefore, the current focus was not yet on finding the best machine learning technique to predict bioimpedance and we used all the data for computing the linear mixed-effect models and neural networks. Still, we also want to evaluate whether the linearity assumption in linear mixed-effect models is appropriated for the context of our study.

#### Muscle force segmentation

We segmented each subject’s signals into twelve different levels of muscle force estimation to study if changes in muscle activity alter bioimpedance signals. The intention of segmenting the data was to study the contributions of homogeneous cycles in terms of muscle force. We tested different number of segmentations and dividing in twelve levels permitted to have a proper resolution in muscle force estimation and sufficient samples to compute the linear models and neural networks. The levels were selected by proportional quantiles of the muscle force values computed for each subject, specifically, the quantiles used as threshold for the segmentations were: 0.08, 0.17, 0.25, 0.33, 0.42, 0.50, 0.67, 0.75, 0.83 and 0.92. In this way we got approximately the same number of cycles and samples per each segment of the data (~11 000 samples corresponding to 55 s). Thus, around 130 000 samples (650 s) were used to compute the linear models and 11 000 for each neural network.

#### Linear models

Linear mixed-effect models were computed using the *acc*_*CM*_ or volume signals as predictor variables, and bioimpedance as response variable (Fig. 2a). These models are an extension of linear regressions which allow the use of longitudinal, multilevel and non-independent data. The level of muscle force was used as grouping variable to adapt the model coefficients to different levels of muscle activity. Three different linear models were computed for each subject and electrode configuration by changing the predictor variables (*acc*_*CM*_ signals, volume or both types). We fitted the linear models using the maximum likelihood estimation.

#### Neural networks

The neural network analysis was included to examine the non-linear relation between bioimpedance and *acc*_*CM*_ signals or volume (Fig. 2b). Along the same lines as linear models we computed three different feedforward neural networks for each subject, level of muscle force and electrode configuration by changing the networks inputs: *acc*_*CM*_ signals, volume or both types. Thus, the input sizes were three, one and four, respectively. The neural networks had one hidden layer of 10 units with hyperbolic tangent sigmoid transfer function. The output of the neural networks was one unit corresponding to the bioimpedance signal and its unit transfer function was linear. We used all the data for training and validation (85% and 15% of the samples randomly chosen, respectively) and none for testing. The training algorithm was Levenberg-Marquardt backpropagation.

#### Statistical analysis

The mean absolute percentage error (MAPE) was used to measure the performance of the linear models and neural networks. The MAPE values were computed between the bioimpedance signals and the output of the linear models and neural networks. The fitted bioimpedance signals were obtained with the same data we used to compute and train the linear mixed-effect models and neural networks to evaluate the adjustment of the data. The MAPE values were calculated cycle by cycle related to the peak-to-peak bioimpedance amplitude of the cycle as follows,

1 $$MAP{E}_{i}( \% )=100\frac{1}{N}\,\mathop{\sum }\limits_{n=1}^{N}\,|\frac{{Z}_{i}[n]-\widehat{{Z}_{i}}[n]}{{Z}_{i}^{PP}[n]}|$$

where ${Z}_{i}[n]$ and $\widehat{{Z}_{i}}[n]$ are the true and the fitted bioimpedance signals during the cycle *i*, *N* is the number of samples and ${Z}_{i}^{PP}[n]$ the bioimpedance peak-to-peak amplitude of the cycle *i*. The main analysis was done with MAPE to quantify the errors related to the amplitude of each cycle. In that way, we prevented the error from varying due to changes in signal amplitude.

Root mean squared errors (RMSE) were also calculated to evaluate the performance of the different techniques using an absolute measure.

The signal processing and data analysis were developed in MATLAB environment (v. R2018a, Natick, MA, USA).

## Results

Bioimpedance, volume and accelerometer data were acquired in ten healthy subjects during an incremental inspiratory threshold protocol. We studied bioimpedance signals by reconstructing it through linear mixed-effect models and neural networks with volume and/or accelerometer signals. The performance was evaluated by the MAPE values obtained from the linear models and neural networks.

### Waveform changes during inspiratory loading

**Figure 3:**
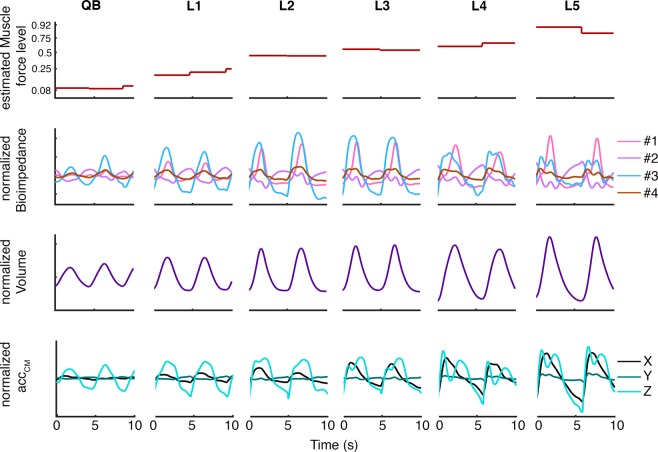
Temporal representation of the signals used in the study: muscle force level, four electrode configuration of bioimpedance, respiratory volume and *acc*_*CM*_. The muscle force level is represented in quantiles of all subject’s values. The other signals were normalized to be in the range of [−1, 1]. #1, #2, #3 and #4 denote the electrode configurations (Fig. 1b). X, Y and Z are the three spatial signals of *acc*_*CM*_. QB, L1, L2, L3, L4 and L5 denote the imposed threshold inspiratory loads. The signals corresponded to subject 2, who illustrated better the bioimpedance changes when the level of estimated muscle force increased.

The signals used in the presented study are represented in Fig. 3 for all loads for subject 2 (S02). The signals of S02 showed large changes during the imposed loads, which illustrate the waveform changes of the bioimpedance signal clearly. Firstly, Fig. 3 shows the increase of muscle force estimation level (RMS |sMMG_*lic*_|) over the loads, from 0.08 in QB to 0.92 in the highest load. The units shown in the muscle force representation are the corresponding quantile values. When high loads were imposed to S02, bioimpedance signal exhibited changes in waveform and temporal behaviour in all the configurations. Apart from the amplitude changes, bioimpedance signal showed waveform changes like the appearance of high frequency content during the highest inspiratory loads. In the temporal point of view, the configurations were no longer in phase, like configurations #1 and #3. The three *acc*_*CM*_ signals changed during the loads in a very similar way as bioimpedance waveform. The appearance of the high frequency content that is visible in the bioimpedance signal can also be observed clearly in the Z component. In contrast, volume waveform did not change, but the subjects inhaled more air when the loads were imposed. In short, Fig. 3 exhibits clear changes in the signals under study when the inspiratory loads were imposed and were more notable when load increased.

### Models performance

#### Volume and chest movement combinations to characterize thoracic bioimpedance

**Figure 4:**
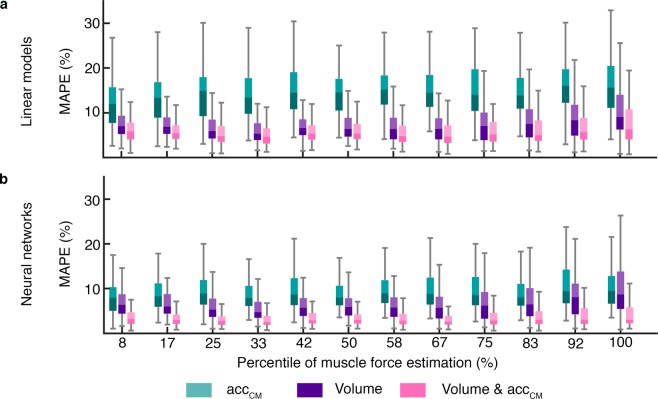
Mean Absolute Percentage Errors between bioimpedance and the fitted bioimpedance for different levels of muscle force estimation. MAPE values from the resulting outputs of (**a**) linear models and (**b**) neural networks. The errors correspond to the different inputs combinations: *acc*_*CM*_, volume, and volume and *acc*_*CM*_. The MAPE values are from electrode configuration #4 which electrodes were the most separated and covered a broader zone.

The MAPE values were computed for each respiratory cycle and all the linear models and neural networks. Figure 4 shows the MAPE values distribution of all subjects’ cycles for the different inputs combinations corresponding to electrode configuration 4. Only *acc*_*CM*_ signals as inputs resulted in higher MAPE values than only volume or both *acc*_*CM*_ and volume inputs. Notice that for linear models the median of the error values were lower than 9.05% when only volume were used, 16.01% when *acc*_*CM*_ signals were used, and 6.31% in case of both signals. However, neural networks clearly presented lower errors, being the median of the MAPE 9.49% when *acc*_*CM*_ signals were used, 8.67% when only were used and 3.02% in case of both signals. Therefore, in both methods when volume and *acc*_*CM*_ signals were used as inputs, the median of MAPE values were always lower than using only volume. Consequently, both linear models and neural networks showed the lowest errors when volume and *acc*_*CM*_ signals were used.

**Figure 5:**
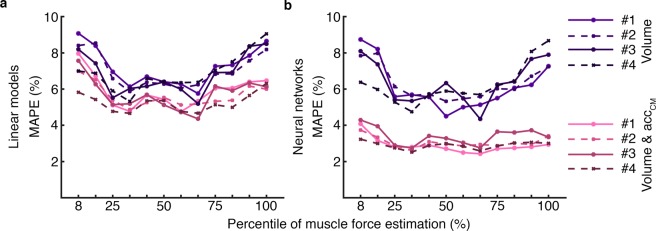
Median values of the Mean Absolute Percentage Errors between bioimpedance and the fitted bioimpedance for different levels of muscle force estimation. The medians values were computed using the resulting outputs of (**a**) linear models and (**b**) neural networks. The errors correspond to the different inputs combinations: *acc*_*CM*_, volume, and volume and *acc*_*CM*_. The four electrode configurations are represented, #1, #2, #3 and #4 (Fig. 1b).

The MAPE values from the linear models and neural networks are shown in Fig. 5 when only volume and both volume and *acc*_*CM*_ were used. Figure 5 depicts the better performance of neural networks when the inputs of the networks included volume and *acc*_*CM*_ signals. For the neural networks using both signals as inputs, the MAPE values remained approximately constant over the different muscle force levels and the median below 4.29% whereas the other models gave higher errors during low and high activity.

**Figure 6:**
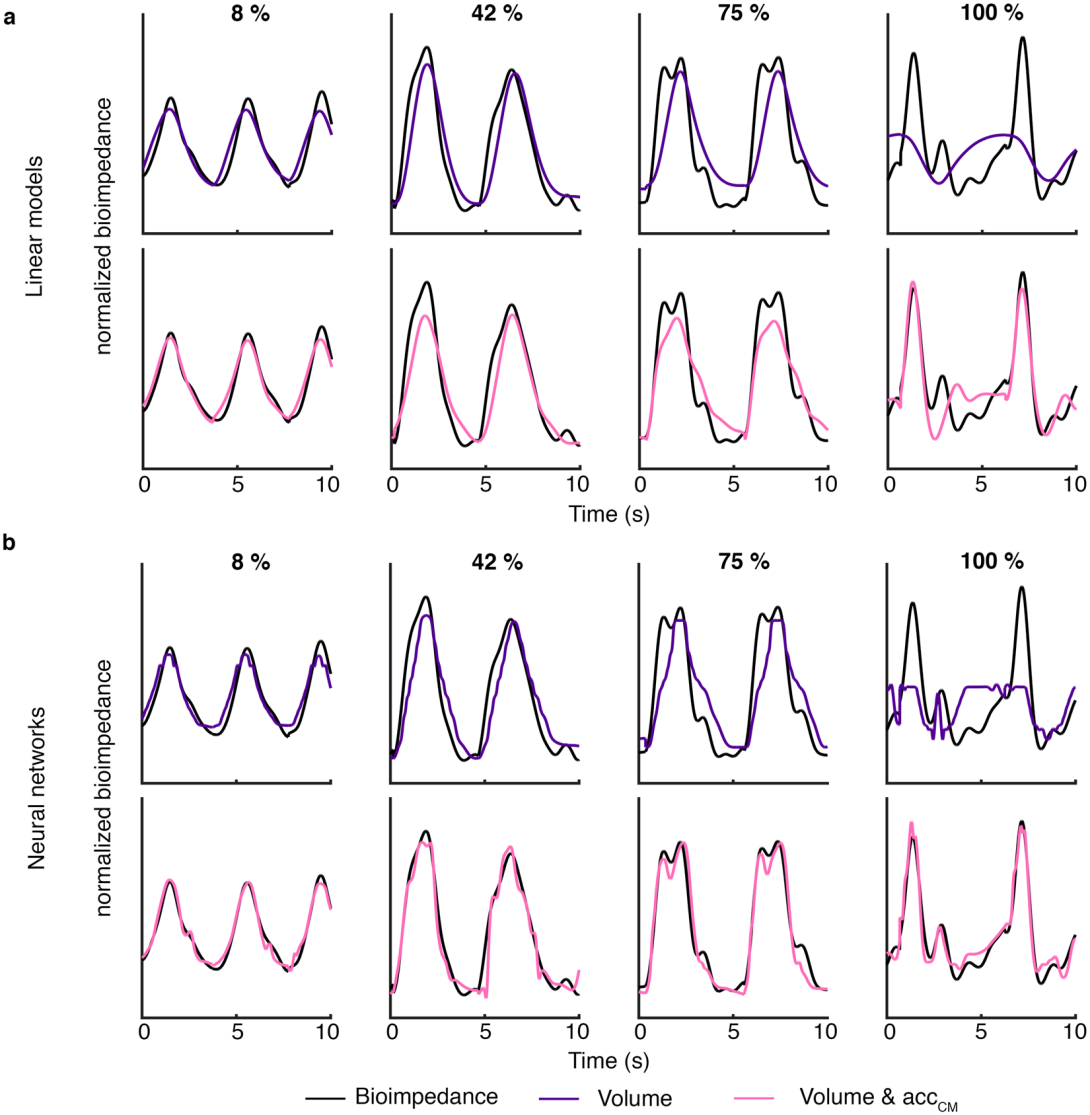
Bioimpedance signal and its corresponding fitted bioimpedance signal examples for 8%, 42%, 75% and 100% of muscle force level. The fitted signals examples were obtained from (**a**) the linear models and (**b**) the neural networks. The examples corresponded to subject 2 and electrode configuration #4 which electrodes were the most separated and covered a broader zone.

The target and fitted bioimpedance signals are shown in Fig. 6 for S02. These examples correspond to 10 seconds of four different data segments, 8%, 42%, 75% and 100% percentile of muscle force estimation. We observed better adjustment between the target and the fitted signals when the techniques used *acc*_*CM*_ and volume as inputs. The best adjustment was observed for the outputs of the neural networks accordingly to the error results. Note that the adjustment improvement is more notable at high muscle activity (75% or 100% percentile).

#### Electrode configurations

**Figure 7:**
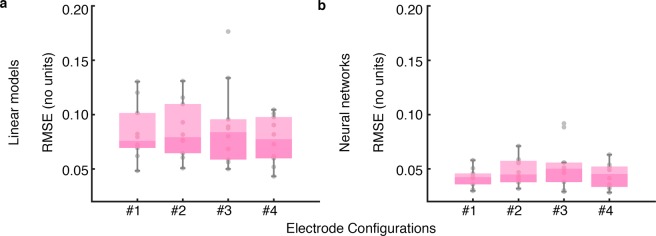
Root Mean Squared Errors between bioimpedance signal and the corresponding fitted bioimpedance signals. The errors were computed for the (**a**) linear models and (**b**) the neural networks when volume and *acc*_*CM*_ signals were used. The represented points correspond to each subject’s error for all electrode configurations. #1, #2, #3 and #4 denote the electrode configurations shown in Fig. 1b.

The performances of the four electrode configurations were similar as Fig. 5 shows. In addition, RMSE values of the entire signals are shown in Fig. 7 for the four configurations. These errors were computed from each subject’s bioimpedance and the corresponding fitted signals when volume and *acc*_*CM*_ signals were used together as inputs. The signals range was [−1, 1] because bioimpedance were normalized in that range. Note that RMSE values were lower for the neural networks than for the linear models which is the same as we observed in MAPE values. All four configurations presented RMSE values approximately in the same range but for neural networks, the median RMSE of configuration 1 was lower than the other configurations.

## Discussion

The objective of the study was to examine the relevance of respiratory volume and chest movement contributions to thoracic bioimpedance. Thoracic bioimpedance and respiratory volume were compared in several studies^5–11^, but most of these studies were under normal breathing. Therefore, our aim was to better understand thoracic bioimpedance changes in relation with both volume and breathing movement under restrictive breathing to support its use for healthcare respiratory monitoring. Hereto, bioimpedance from four electrode configurations, respiratory airflow and accelerometer data were measured in ten healthy subjects during an inspiratory threshold loading protocol. This protocol allowed us to analyse the impact of these contributions during changes in breathing pattern and muscle force. We reconstructed the bioimpedance signals from volume, chest movement and the combination of both using linear mixed-effect models and neural networks. The comparison between the actual and the fitted bioimpedance signals permitted to evaluate the relevance of the volume and chest movement contributions. The main novelty is the inclusion of chest movement in the characterization of thoracic bioimpedance measurement for different levels of muscle force.

The performance of the techniques was measured with the MAPE computed for each cycle. Lower errors were obtained when the linear models and neural networks were computed using the volume and *acc*_*CM*_ signals (Figs. 4 and 5). In linear models when only volume was used, the median of the MAPE were below 9.08% whereas in neural networks the median of values were below 8.74%. On the other hand, when the *acc*_*CM*_ signals were added to the models the median of the MAPE were below 7.94% for linear models and 4.29% for neural networks. Therefore, the MAPE values obtained from both techniques were good and improved when *acc*_*CM*_ signals were included. These results exhibited that linear models could be used to approximate the contributions of volume and chest movement to thoracic bioimpedance but neural networks described the relation even better. On the other hand, the median errors with only volume were lower than the ones when only *acc*_*CM*_ signals were used in linear models and neural networks for all muscle force levels (Fig. 4). Therefore, the contribution of volume was crucial for a good explanation of bioimpedance changes.

The relationship between bioimpedance and respiratory volume was studied previously and a linear relation between them was reported^5–11^. Focusing only on the relationship between bioimpedance and volume, we observed in Figs. 4 and 5 that the errors of linear models and neural networks are practically in the same range which may mean that the relation was essentially linear. These results are comparable to the ones of Baker *et al*.^29^ who computed linear and non-linear regressions to characterize different electrode configurations of bioimpedance using respiratory volume. They found that fourth degree polynomial regression characterized the data better but the differences with linear regressions were often small.

Recent studies described nonlinear relations between bioimpedance and respiratory volume during abnormal breathing like maximal respiratory maneuvers and airway obstructions^8,10^. Therefore, the non-linearity showed in these studies seems to be related to the breathing pattern and mechanics of the subjects’ breathing. From these studies we deduced that the relationship between bioimpedance and volume is dependent on the electrodes location and the way the subjects breathed in increased breathing effort conditions. We hypothesized that the nonlinear relationship between bioimpedance and volume can be explained as changes in the impedance contributions. Along these lines, we included the chest movement as a contribution of thoracic bioimpedance to analyze if it can be related to the apparent non-linearity. We found a better performance in the characterizations which used volume and *acc*_*CM*_ (Fig. 4). Particularly, neural networks clearly showed an improvement over linear models thus the chest movement contribution was described better as nonlinear. The nonlinearity is difficult to characterize previously because it is subject dependent. However, neural networks allowed us to analyze the nonlinear relations without establishing them previously. Not many bioimpedance studies included neural networks to their respiratory research. Młyńczak *et al*. used neural networks but for nonlinear calibrations. They found better accuracy in nonlinear calibration with neural networks than with simple linear modeling^30^.

Regarding to the electrode configurations, the RMSE obtained from the target and fitted bioimpedance signals was slightly smaller for configuration 1 (Fig. 7). These results agree with our previous study in which all configurations presented similar results but configuration 1 exhibited a robust performance in terms of concordance with volume^11^. Although in terms of error all configurations were quite similar, we observed different behavior in the signal waveform from the electrode configurations as Fig. 3 shows. This is consistent our previous temporal analysis of bioimpedance and volume in which delays were observed between the signals^21^. These delays were dependent on bioimpedance electrode location and changed with loads. Hence, none of the four configuration performed clearly better but we observed differences in signal waveform. These waveform differences can be related to the different impedance contributions of the zones covered by the electrode configurations.

In the presented study, accelerometer data were used for two different purposes, as measure of chest movement and as estimation of muscle force. Previous studies suggested that accelerometer data in the high frequency band (|sMMG_*lic*_|) is related to the inspiratory effort^20,27,31^. Lozano-García *et al*. found strong correlation between |sMMG_*lic*_| and the inspiratory muscle function in healthy subjects and during the same loading protocol as the presented study^20^. Therefore, |sMMG_*lic*_| permitted us to divide the respiratory cycles into different levels of muscle force estimation.

We divided the respiratory cycles into twelve levels of muscle force estimation selected by proportional quantiles. Figure 5 shows the medians of the MAPE values for each level of activity when volume and volume in combination with *acc*_*CM*_ were used. Higher errors were observed for the extreme levels of muscle activity in each method except when neural networks included volume and chest movement. The lower levels corresponded to the quiet breathing cycles in which the amplitude of the signals is lower (Fig. 3) so the corresponding MAPE values were related to lower peak-to-peak amplitudes and consequently higher. On the contrary, at high level of muscle activity the higher error values were due to a lower performance in the characterization of bioimpedance. In particular, this increase in error when only respiratory volume was used, was likely because volume did not explain completely bioimpedance especially during high muscle force level. In addition, the increase of error in the linear models with volume and *acc*_*CM*_ was probably due to the non-linearity between the signals. On the other hand, the neural networks with volume and *acc*_*CM*_ as inputs exhibited a better performance since the errors were lower than the other methods for all levels of muscle force. This better performance can be also observed in the comparison between the target and fitted bioimpedance signals of Fig. 6. Contrary to the other characterizations which performance worsen at high levels of muscle force, the performance of this method practically did not vary. Consequently, the relation between bioimpedance and volume can be basically described as linear. However, the addition of the chest movement improved the characterizations especially in neural networks and for high levels of muscle force. Therefore, the chest movement contribution was more relevant for high muscle activity than for middle activity.

The results from this study suggest that the combination of thoracic bioimpedance and chest movement could be promising for respiratory monitoring. The combination of the two signals could lead to an improved volume prediction. It becomes even more relevant during restrictive breathing, which is common in respiratory patients. Following the results from this study, further studies including thoracic bioimpedance and accelerometer signals should validate the suitability of these signals to predict volume. Hereto, larger databases including patients with pulmonary diseases will be needed to reinforce the use of these physiological signals in clinical application.

In summary, we investigated the relevance of volume and chest movement to thoracic bioimpedance at different levels of muscle force. In accordance to previous studies, we showed that the relation between volume and thoracic bioimpedance was essentially linear which supports the clinical application of bioimpedance for respiratory monitoring. However, the presented results exhibited that the combination of respiratory volume and *acc*_*CM*_ characterized better the thoracic bioimpedance measurement for all levels of muscle activity. The linear approximation showed good results although neural networks described better the volume and chest movement contributions to bioimpedance. We did not find substantial differences in electrode configurations which means that all four included volume and chest movement contributions. Accordingly, we conclude that thoracic bioimpedance changes were fundamentally due to the respiratory volume, although chest movement contributed substantially to bioimpedance measurement and its contribution was more relevant at high muscle activity levels. Finally, the presented results provided a better understanding of the changes of thoracic bioimpedance measurement and its relation with muscle activity changes. Our contribution will help in the application of thoracic bioimpedance and accelerometer data as a non-invasive healthcare technique for respiratory monitoring.

## Data Availability

The dataset analysed during the current study is available from the corresponding author on reasonable request.
